# Early mortality risk prediction in critically ill patients with liver cancer using interpretable machine learning: a retrospective cohort study

**DOI:** 10.3389/fcell.2026.1882859

**Published:** 2026-07-06

**Authors:** Qin Li, Bo Zhang, Lei Zhang, Jiawei Luo, Shixin Huang

**Affiliations:** 1 Infectious Disease Department, Mianyang 404 Hospital, Mianyang, China; 2 Department of Radiology, Mianyang Fulin Hospital, Mianyang, China; 3 School of Humanities and Social Sciences, Neijiang Health Vocational College, Neijiang, China; 4 Evidence and Guideline Excellence Collaborative Innovation Laboratory, Children’s Hospital of Chongqing Medical University, Chongqing, China; 5 Department of Scientific Research, The People’s Hospital of Yubei District of Chongqing City, Chongqing, China

**Keywords:** 28-day mortality, acute kidney injury, intensive care unit, liver cancer, machine learning, malignancy, random forest, shap

## Abstract

**Objective:**

This study aimed to develop and internally evaluate interpretable machine learning models for predicting 28-day all-cause mortality among patients with liver cancer admitted to the intensive care unit (ICU).

**Methods:**

A total of 507 ICU patients with liver cancer were identified from the Medical Information Mart for Intensive Care IV database. Patients were randomly divided into a training set and a test set at a ratio of 7:3 using stratified sampling. Candidate predictors were restricted to variables available at ICU admission or within the first 24 h after ICU admission. Feature selection was performed using clinical screening, collinearity assessment, and least absolute shrinkage and selection operator regression. Four models were developed, including logistic regression (LR), least absolute shrinkage and selection operator logistic regression (LASSO logistic regression), random forest (RF), and extreme gradient boosting (XGBoost). Model performance was evaluated using AUROC, AUPRC, Brier score, sensitivity, specificity, positive predictive value, negative predictive value, and F1 score. SHapley Additive exPlanations were used to interpret the best-performing model. A simplified model based on key predictors was also constructed.

**Results:**

Among the 507 included patients, 150 patients died within 28 days after ICU admission, corresponding to a mortality rate of 29.6%. The random forest model achieved the best performance, with an AUROC of 0.974, AUPRC of 0.950, Brier score of 0.065, sensitivity of 84.4%, specificity of 98.1%, and F1 score of 0.894. It outperformed logistic regression, LASSO logistic regression, XGBoost, and conventional severity scores, including SOFA and MELD. SHAP analysis identified APS III, Charlson Comorbidity Index, MELD, blood urea nitrogen, and glucose as important predictors. The simplified 12-variable model retained good discrimination, with an AUROC of 0.890.

**Conclusion:**

An interpretable random forest model showed high internal predictive performance for 28-day mortality among ICU patients with liver cancer. External and prospective validation is required before clinical implementation.

## 1 Background

Liver cancer, mainly including hepatocellular carcinoma (HCC) and intrahepatic cholangiocarcinoma (ICC), remains one of the most lethal malignancies worldwide. It ranks as the sixth most commonly diagnosed cancer and the third leading cause of cancer-related mortality, with approximately 905,000 new cases and 830,000 deaths each year (Sung et al., 2021; Llovet et al., 2021). Patients with advanced liver cancer who require intensive care unit (ICU) admission represent a particularly vulnerable group. These patients often present with decompensated cirrhosis, coagulation disorders, portal hypertension, and multi-organ dysfunction (Forner et al., 2018; Berzigotti et al., 2013). Although reported ICU mortality among patients with liver cancer ranges from 25% to 50%, prognostic tools tailored to this specific population remain insufficient (McPhail et al., 2015).

Traditional ICU severity scores, such as SOFA, SAPS II, APS III, and OASIS, were originally developed for broad critically ill populations rather than for patients with liver cancer (Vincent et al., 1996; Le Gall et al., 1993; Knaus et al., 1985; Zimmerman et al., 2006; Johnson et al., 2013). These scores may not fully reflect the complex risk profile of this subgroup. In particular, they may insufficiently capture the combined hepatic, oncological, and systemic burden observed in critically ill patients with liver cancer. Similarly, the Model for End-Stage Liver Disease (MELD) has been widely validated for assessing end-stage liver disease. However, it does not directly incorporate tumor burden, cancer-related complications, or sepsis-associated organ dysfunction. These factors are common among ICU patients with liver cancer (Kamath and Kim, 2007). Therefore, more accurate and clinically relevant risk stratification approaches are needed for this high-risk population.

Machine learning (ML) approaches have increasingly been used for clinical outcome prediction. These approaches can model complex and non-linear relationships among multidimensional clinical variables (Handelman et al., 2018). The use of ML in critical care research has expanded alongside open critical care datasets and clinical data-science tools (Johnson et al., 2016), and ML-based approaches have been applied to sepsis-related risk prediction (Shukeri et al., 2018) and early prediction of acute respiratory distress syndrome (Le et al., 2020). However, evidence regarding ML-based mortality prediction specifically for ICU patients with liver cancer remains limited. In particular, interpretable ML models for early 28-day mortality prediction in this population remain insufficiently investigated.

MIMIC-IV is a publicly available critical care database that contains detailed demographic, laboratory, treatment, and physiological information from more than 73,000 ICU admissions at a large academic medical center (Johnson et al., 2023). Its high-resolution clinical data make it suitable for developing prediction models in less-studied ICU subgroups, including patients with liver cancer.

This study aimed to: (1) develop and internally evaluate 4 ML models, including logistic regression (LR), LASSO logistic regression, random forest (RF), and XGBoost, for predicting 28-day all-cause mortality among ICU patients with liver cancer using MIMIC-IV; (2) compare these models with five conventional clinical scoring systems; (3) use SHAP to interpret the best-performing model; (4) construct a simplified model based on the top SHAP-ranked predictors; and (5) conduct Kaplan-Meier survival analysis, subgroup analysis, and sensitivity analysis to assess model robustness.

## 2 Methods

### 2.1 Data source and study population

**FIGURE 1:**
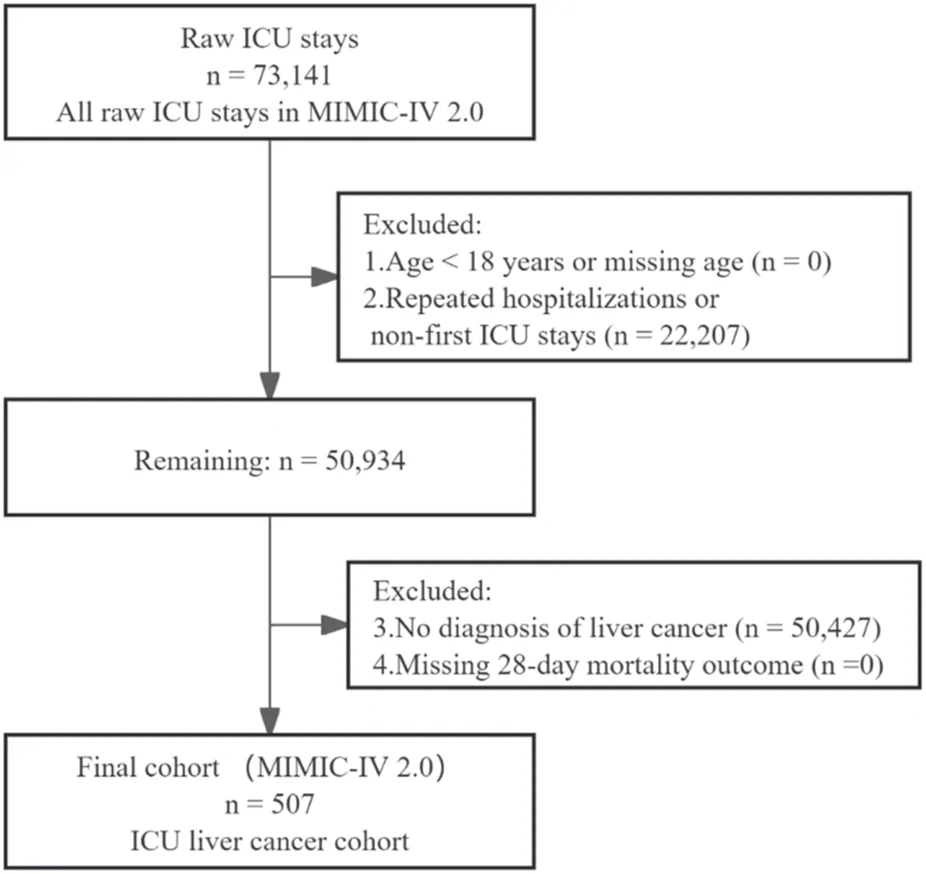
Patient selection flowchart. Stepwise inclusion and exclusion criteria applied to MIMIC-IV ICU admissions to derive the final analytic cohort of 507 patients with liver cancer.

Data were obtained from MIMIC-IV version 2.2, a single-center retrospective critical care database maintained by Beth Israel Deaconess Medical Center in Boston, Massachusetts, USA (Johnson et al., 2023). Liver cancer was identified using ICD-10-CM codes C22.0, C22.1, and C22.9, together with corresponding ICD-9 codes 155.0, 155.1, and 155.2. These codes covered HCC, ICC, and other primary hepatic malignancies. Patients were included if they met the following criteria: (1) age ≥18 years; (2) first ICU stay during the first hospitalization; and (3) available information on 28-day mortality. Repeated hospital admissions and repeated ICU stays were excluded to reduce within-patient correlation and ensure independent observations. The patient selection process is shown in Figure 1.

The primary outcome was 28-day all-cause mortality, defined as death occurring within 28 days after ICU admission. To reflect early clinical decision-making, all candidate predictors were restricted to variables available at ICU admission or within the first 24 h after ICU admission.

### 2.2 Variable extraction

Candidate variables were extracted from the following domains: (1) demographic characteristics, including age, sex, and ethnicity; (2) liver cancer type, including HCC, ICC, and liver metastasis; (3) liver disease-related conditions, including cirrhosis, viral hepatitis B or C, alcoholic liver disease, liver failure, ascites, hepatic encephalopathy, portal hypertension, and coagulopathy; (4) comorbidities, including Charlson Comorbidity Index (CCI), metastatic solid tumor, renal disease, congestive heart failure, and chronic kidney disease (Quan et al., 2011); (5) vital signs within the first 24 h after ICU admission, including heart rate, systolic blood pressure, diastolic blood pressure, mean arterial pressure, respiratory rate, temperature, oxygen saturation, and Glasgow Coma Scale; (6) laboratory tests within the first 24 h, including lactate, creatinine, blood urea nitrogen (BUN), total bilirubin, albumin, international normalized ratio (INR), prothrombin time, platelet count, hemoglobin, alanine aminotransferase (ALT), aspartate aminotransferase (AST), alkaline phosphatase (ALP), glucose, sodium, potassium, bicarbonate, anion gap, PaO_2_, PaCO_2_, pH, and base excess; (7) organ support therapies, including mechanical ventilation, invasive or non-invasive ventilation, vasopressor use, renal replacement therapy (RRT), and urine output; and (8) severity scores, including SOFA, Simplified Acute Physiology Score II (SAPS II), APS III, Oxford Acute Severity of Illness Score (OASIS), MELD, and Logistic Organ Dysfunction Score. Variables with a missing rate >40% in the training set were excluded from model development.

### 2.3 Data preprocessing and feature selection

The dataset was divided into a training set and a test set at a ratio of 70:30 using stratified random sampling based on 28-day mortality status. The training set included 354 patients, and the test set included 153 patients. A fixed random seed was used for reproducibility. To avoid data leakage, all preprocessing steps were fitted only on the training set and then applied to the test set.

Continuous variables were imputed using the median value from the training set, whereas categorical variables were imputed using the mode from the training set. Categorical variables were transformed using one-hot encoding. Continuous variables were standardized with StandardScaler for LR and LASSO logistic regression, whereas tree-based models were trained using the original unstandardized values. Class imbalance, with an overall mortality rate of approximately 29.6%, was addressed by applying class_weight = ‘balanced’ for LR and RF and scale_pos_weight for XGBoost. The class_weight = ‘balanced’ parameter adjusts class weights inversely proportional to class frequencies and was used to reduce the influence of outcome imbalance during model training. SMOTE was not used in the primary analysis.

**FIGURE 2:**
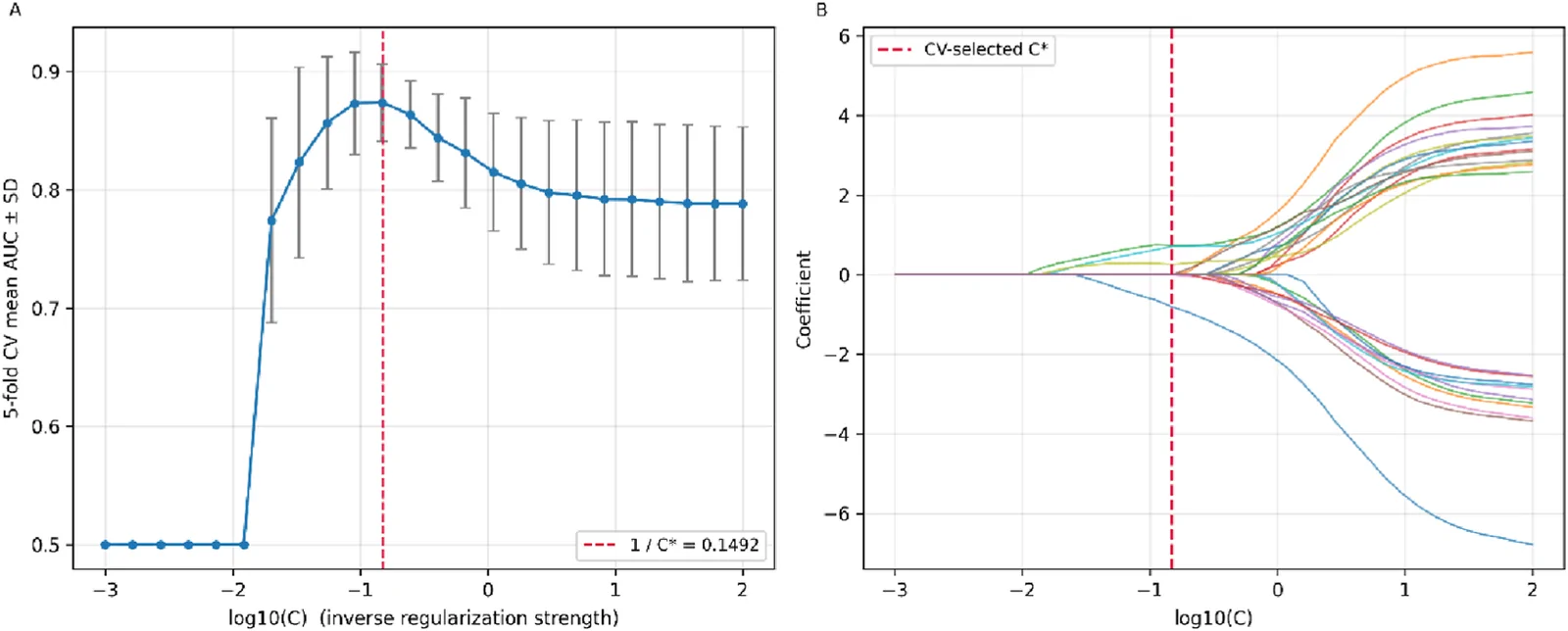
LASSO feature selection. **(A)** Cross-validation error curve according to the regularization parameter λ; dashed vertical lines indicate λ.min and λ.1se. **(B)** Coefficient regularization path showing the shrinkage of candidate predictors toward zero.

Feature selection was performed in three steps. First, clinically relevant variables were retained based on pathophysiological plausibility and availability within the first 24 h after ICU admission. Second, Pearson correlation analysis was used to reduce collinearity, and one variable was removed from each pair with an absolute correlation coefficient ≥0.85. Third, LASSO logistic regression with 5-fold cross-validation was applied to identify variables with non-zero coefficients, following established regularized regression methods for variable selection (Tibshirani, 1996; Zou and Hastie, 2005). The final full model included 22 selected predictors. The LASSO cross-validation curve and coefficient regularization path are presented in Figure 2.

### 2.4 Machine learning model development

Four ML models were developed: LR with L2 regularization, LASSO logistic regression with L1 regularization, RF, and XGBoost (Breiman, 2001; Chen and Guestrin, 2016). Hyperparameters were optimized using 5-fold stratified cross-validation within the training set. The independent test set was reserved exclusively for final model evaluation and was not used during model tuning. All trained models were saved using joblib to support reproducibility.

### 2.5 Model evaluation

Model performance in the test set was evaluated using AUROC, AUPRC, Brier score, accuracy, sensitivity, specificity, PPV, NPV, and F1 score. The optimal classification threshold was determined using the Youden index in the training set and was then directly applied to the test set without further adjustment. Bootstrap resampling with 1,000 iterations was used to calculate 95% CIs for AUROC, AUPRC, and Brier score (Tibshirani and Efron, 1993). The Brier score was used to quantify probabilistic prediction error (Glenn, 1950), while AUROC-based discrimination was interpreted according to standard ROC analysis methodology (DeLong et al., 1988). Calibration performance was assessed using the calibration slope and intercept. Decision curve analysis (DCA) was performed to estimate net clinical benefit across a range of threshold probabilities (Vick et al., 2006).

Five conventional clinical scoring systems, including SOFA, SAPS II, APS III, MELD, and OASIS, were evaluated as univariate predictors in the test set and compared with the ML models.

### 2.6 Model interpretability and simplified model

SHAP with TreeExplainer, a SHAP explainer specifically adapted for tree-based models, was applied to the best-performing RF model to quantify the contribution of each predictor to the model output (Lundberg and Lee, 2017; Lundberg et al., 2020). Global feature importance was determined using mean absolute SHAP values. SHAP dependence plots were generated for the leading predictors to examine their relationships with predicted risk. In addition, waterfall plots were used to illustrate individual-level predictions for two representative patients, including one high-risk patient who died within 28 days and one low-risk patient who survived beyond 28 days.

To improve clinical usability, a simplified model was constructed using the top 12 SHAP-ranked predictors after excluding variables with potential outcome leakage. This simplified model was trained using the same RF algorithm and hyperparameter settings and was evaluated in the same test set.

### 2.7 Survival and additional analyses

Kaplan-Meier survival curves were generated for all 4 ML models (Kaplan and Meier, 1958). Patients in the test set were classified into high-risk and low-risk groups according to the Youden-index thresholds derived from the training set. Log-rank tests were used to compare survival distributions between risk groups.

Univariate and multivariate Cox proportional hazards regression analyses were performed in the full cohort using key clinical variables (Cox, 1972). The proportional hazards assumption was assessed using scaled Schoenfeld residuals (Grambsch and Therneau, 1994). Subgroup analyses were conducted to evaluate RF performance in five predefined clinical subgroups: HCC, cirrhosis, sepsis, mechanical ventilation, and vasopressor use.

Five sensitivity analyses were further performed to examine the robustness of the model: (SA1) exclusion of patients who died within 24 h after ICU admission; (SA2) restriction to patients with primary HCC; (SA3) removal of composite severity score variables followed by model retraining; (SA4) complete-case analysis; and (SA5) repeated model training using five different random seeds.

### 2.8 Statistical analysis

Continuous variables are expressed as mean ± standard deviation (SD) for normally distributed data or as median with interquartile range (IQR) for non-normally distributed data. Normality was assessed using the Shapiro-Wilk test. Between-group comparisons were performed using Student’s t-test or the Mann-Whitney U test for continuous variables, and the chi-square test or Fisher’s exact test for categorical variables, as appropriate. A two-sided p value <0.05 was considered statistically significant. All analyses were conducted using Python 3.9 with scikit-learn, lifelines, SHAP, and statsmodels (Pedregosa et al., 2011).

### 2.9 Ethics

This study was based on de-identified data from MIMIC-IV, which is available for approved research use. Because all patient information in MIMIC-IV is de-identified, additional institutional review board approval was not required for this secondary analysis. All data access and analyses complied with the relevant data use agreements.

## 3 Results

### 3.1 Patient selection and baseline characteristics

After applying the predefined inclusion and exclusion criteria, 507 ICU patients with liver cancer were included in the final analytic cohort (Figure 1). Among them, 150 patients (29.6%) died within 28 days after ICU admission. The training set included 354 patients, of whom 105 died within 28 days, corresponding to a mortality rate of 29.7%. The test set included 153 patients, with 45 deaths and a 28-day mortality rate of 29.4%.

Baseline characteristics according to 28-day mortality status are summarized in Table 1. Survivors and non-survivors were generally comparable in age (64.27 ± 10.85 years vs. 65.41 ± 12.18 years; p = 0.158) and sex distribution (p = 0.221). However, several clinically meaningful differences were observed between the two groups. Compared with survivors, non-survivors had higher proportions of liver failure (46.0% vs. 16.2%; p < 0.001), ascites (42.0% vs. 19.9%; p < 0.001), hepatic encephalopathy (28.7% vs. 10.1%; p < 0.001), coagulopathy (25.3% vs. 10.1%; p < 0.001), hepatitis B infection (16.7% vs. 9.0%; p = 0.012), alcoholic liver disease (31.3% vs. 22.1%; p = 0.029), and metastatic solid tumor (40.7% vs. 17.1%; p < 0.001). The Charlson Comorbidity Index (CCI) was also significantly higher among non-survivors than among survivors (9.33 ± 2.75 vs. 7.45 ± 2.37; p < 0.001).

**TABLE 1:** Baseline characteristics of ICU patients with liver cancer according to 28-day mortality.

Characteristics	Total (N = 507)	Survivors (N = 357)	Non-survivors (N = 150)	*p*-value
Demographics				
Age (years)	64.61 ± 11.26	64.27 ± 10.85	65.41 ± 12.18	0.158
Male sex, *n* (%)	384 (75.7)	265 (74.2)	119 (79.3)	0.221
Liver cancer type				
HCC, *n* (%)	379 (74.8)	263 (73.7)	116 (77.3)	0.386
ICC, *n* (%)	115 (22.7)	86 (24.1)	29 (19.3)	0.243
Metastatic solid tumor, *n* (%)	122 (24.1)	61 (17.1)	61 (40.7)	<0.001
Liver disease				
Cirrhosis, *n* (%)	332 (65.5)	228 (63.9)	104 (69.3)	0.237
Hepatitis B, *n* (%)	57 (11.2)	32 (9.0)	25 (16.7)	0.012
Hepatitis C, *n* (%)	178 (35.1)	133 (37.3)	45 (30.0)	0.118
Alcoholic liver disease, *n* (%)	126 (24.9)	79 (22.1)	47 (31.3)	0.029
Liver failure, *n* (%)	127 (25.0)	58 (16.2)	69 (46.0)	<0.001
Ascites, *n* (%)	134 (26.4)	71 (19.9)	63 (42.0)	<0.001
Hepatic encephalopathy, *n* (%)	79 (15.6)	36 (10.1)	43 (28.7)	<0.001
Coagulopathy, *n* (%)	74 (14.6)	36 (10.1)	38 (25.3)	<0.001
Comorbidities				
CCI	8.01 ± 2.63	7.45 ± 2.37	9.33 ± 2.75	<0.001
Renal disease, *n* (%)	79 (15.6)	40 (11.2)	39 (26.0)	<0.001
Severe liver disease, *n* (%)	236 (46.5)	148 (41.5)	88 (58.7)	<0.001
Acute kidney injury, *n* (%)	202 (39.8)	121 (33.9)	81 (54.0)	<0.001
Organ support				
Mechanical ventilation, *n* (%)	346 (68.2)	243 (68.1)	103 (68.7)	0.895
Vasopressor use, *n* (%)	109 (21.5)	56 (15.7)	53 (35.3)	<0.001
RRT, *n* (%)	18 (3.6)	8 (2.2)	10 (6.7)	0.014
Urine output (mL/24 h)	1,353.80 ± 937.10	1,503.62 ± 937.88	989.67 ± 832.02	<0.001
Laboratory tests (first 24 h)				
Lactate, mean (mmol/L)	3.46 ± 3.04	3.07 ± 2.50	4.28 ± 3.80	0.004
Creatinine, max (mg/dL)	1.56 ± 1.19	1.31 ± 1.00	2.16 ± 1.40	<0.001
BUN, max (mg/dL)	32.71 ± 25.58	26.79 ± 20.41	46.97 ± 30.72	<0.001
Total bilirubin, max (mg/dL)	5.70 ± 7.19	4.24 ± 4.88	9.13 ± 10.06	<0.001
Albumin, min (g/dL)	2.81 ± 0.65	2.84 ± 0.59	2.74 ± 0.77	0.126
INR, max	1.78 ± 0.72	1.64 ± 0.55	2.10 ± 0.93	<0.001
Hemoglobin, min (g/dL)	9.50 ± 1.96	9.69 ± 1.86	9.05 ± 2.12	<0.001
Glucose, mean (mg/dL)	153.10 ± 57.49	162.80 ± 57.08	129.68 ± 51.57	<0.001
Severity scores				
SOFA	7.36 ± 4.06	6.53 ± 3.43	9.35 ± 4.73	<0.001
SAPS II	42.23 ± 15.28	37.79 ± 11.90	52.81 ± 17.17	<0.001
APS III	54.55 ± 24.53	46.94 ± 19.07	72.65 ± 26.58	<0.001
MELD	21.57 ± 8.82	19.22 ± 7.64	27.17 ± 8.93	<0.001
OASIS	32.47 ± 8.27	30.93 ± 7.24	36.13 ± 9.38	<0.001
Outcomes				
ICU LOS (days)	3.02 ± 3.76	2.76 ± 3.92	3.63 ± 3.28	<0.001
Hospital LOS (days)	10.91 ± 11.54	12.21 ± 12.92	7.84 ± 6.30	<0.001
28-day mortality, *n* (%)	150 (29.6)	—	—	—

Abbreviations: ICU, intensive care unit; HCC, hepatocellular carcinoma; ICC, intrahepatic cholangiocarcinoma; CCI, charlson comorbidity index; RRT, renal replacement therapy; BUN, blood urea nitrogen; INR, international normalized ratio; SOFA, sequential organ failure assessment; SAPS II, Simplified Acute Physiology Score II; APS III, Acute Physiology Score III; MELD, Model for End-Stage Liver Disease; OASIS, oxford acute severity of illness score; LOS, length of stay.

Laboratory indicators further suggested more severe hepatic and renal dysfunction in the non-survivor group. Non-survivors had higher blood urea nitrogen (BUN; 46.97 ± 30.72 vs. 26.79 ± 20.41 mg/dL; p < 0.001), creatinine (2.16 ± 1.40 vs. 1.31 ± 1.00 mg/dL; p < 0.001), total bilirubin (9.13 ± 10.06 vs. 4.24 ± 4.88 mg/dL; p < 0.001), and international normalized ratio (INR; 2.10 ± 0.93 vs. 1.64 ± 0.55; p < 0.001). In contrast, urine output was significantly lower in non-survivors than in survivors (989.67 ± 832.02 vs. 1,503.62 ± 937.88 mL/24 h; p < 0.001). Conventional severity scores were consistently higher in non-survivors, including SOFA (9.35 ± 4.73 vs. 6.53 ± 3.43; p < 0.001), SAPS II (52.81 ± 17.17 vs. 37.79 ± 11.90; p < 0.001), APS III (72.65 ± 26.58 vs. 46.94 ± 19.07; p < 0.001), MELD (27.17 ± 8.93 vs. 19.22 ± 7.64; p < 0.001), and OASIS (36.13 ± 9.38 vs. 30.93 ± 7.24; p < 0.001). Vasopressor use was also more frequent among non-survivors (35.3% vs. 15.7%; p < 0.001).

### 3.2 Cox regression analysis of key prognostic factors

The results of univariate and multivariate Cox proportional hazards regression analyses are presented in Table 2. In the univariate analysis, several variables were significantly associated with 28-day mortality, including liver failure (hazard ratio [HR] = 3.28, 95% CI: 2.38–4.51; p < 0.001), lactate (HR = 1.11, 95% CI: 1.07–1.15; p < 0.001), INR (HR = 1.91, 95% CI: 1.64–2.21; p < 0.001), vasopressor use (HR = 2.71, 95% CI: 1.94–3.78; p < 0.001), SOFA score (HR = 1.19, 95% CI: 1.15–1.24; p < 0.001), MELD score (HR = 1.12, 95% CI: 1.10–1.14; p < 0.001), CCI (HR = 1.21, 95% CI: 1.15–1.27; p < 0.001), creatinine (HR = 1.39, 95% CI: 1.29–1.50; p < 0.001), and BUN (HR = 1.02; p < 0.001).

**TABLE 2:** Cox proportional hazards regression analysis of key predictors for 28-day mortality in ICU patients with liver cancer.

Variable	Univariate HR (95% CI)	p-value	Multivariate HR (95% CI)	p-value
Age (per 1 year)	1.01 (0.99–1.02)	0.237	1.01 (0.99–1.02)	0.369
Cirrhosis	1.23 (0.88–1.74)	0.225	0.90 (0.61–1.35)	0.617
Liver failure	3.28 (2.38–4.51)	<0.001	1.84 (1.29–2.63)	0.001
Lactate (per 1 mmol/L)	1.11 (1.07–1.15)	<0.001	1.10 (1.05–1.15)	<0.001
Total bilirubin (per 1 mg/dL)	1.06 (1.05–1.08)	<0.001	1.01 (0.98–1.04)	0.45
Albumin (per 1 g/dL)	0.76 (0.55–1.03)	0.08	0.69 (0.53–0.90)	0.007
INR (per 1 unit)	1.91 (1.64–2.21)	<0.001	1.30 (1.01–1.68)	0.046
Platelet (per 1 × 10^9^/L)	1.00 (1.00–1.00)	0.611	1.00 (1.00–1.00)	0.006
Creatinine (per 1 mg/dL)	1.39 (1.29–1.50)	<0.001	1.03 (0.86–1.22)	0.77
BUN (per 1 mg/dL)	1.02 (1.02–1.02)	<0.001	1.01 (1.00–1.02)	0.006
Mechanical ventilation	1.04 (0.74–1.45)	0.839	1.11 (0.78–1.57)	0.568
Vasopressor use	2.71 (1.94–3.78)	<0.001	0.83 (0.53–1.31)	0.427
SOFA (per 1 point)	1.19 (1.15–1.24)	<0.001	1.05 (0.99–1.12)	0.097
MELD (per 1 point)	1.12 (1.10–1.14)	<0.001	1.07 (1.03–1.12)	0.002
CCI (per 1 point)	1.21 (1.15–1.27)	<0.001	1.17 (1.10–1.24)	<0.001

In the multivariate Cox model, eight variables remained independently associated with 28-day mortality. These included liver failure (HR = 1.84, 95% CI: 1.29–2.63; p = 0.001), lactate (HR = 1.10, 95% CI: 1.05–1.15; p < 0.001), albumin (HR = 0.69, 95% CI: 0.53–0.90; p = 0.007), INR (HR = 1.30, 95% CI: 1.01–1.68; p = 0.046), platelet count (HR = 1.00; p = 0.006), BUN (HR = 1.01; p = 0.006), MELD score (HR = 1.07, 95% CI: 1.03–1.12; p = 0.002), and CCI (HR = 1.17, 95% CI: 1.10–1.24; p < 0.001). The proportional hazards assumption was assessed using scaled Schoenfeld residuals, with diagnostic results provided in Supplementary Table S1.

### 3.3 Feature selection

After clinical screening and collinearity filtering using a Pearson correlation threshold of |r| ≥ 0.85, LASSO logistic regression with 5-fold cross-validation was applied for further variable selection. A total of 22 predictors were retained for full model development. The LASSO cross-validation curve and coefficient regularization path are shown in Figure 2. The selected predictors with high ranking included APS III, CCI, minimum respiratory rate, first-measured glucose, MELD-calculated, maximum INR, minimum BUN, and maximum bilirubin. These variables were clinically consistent with the prognostic factors identified in the Cox regression analysis and reflected the combined contribution of acute physiological derangement, liver dysfunction, renal impairment, and comorbidity burden.

### 3.4 Machine learning model performance

**FIGURE 3:**
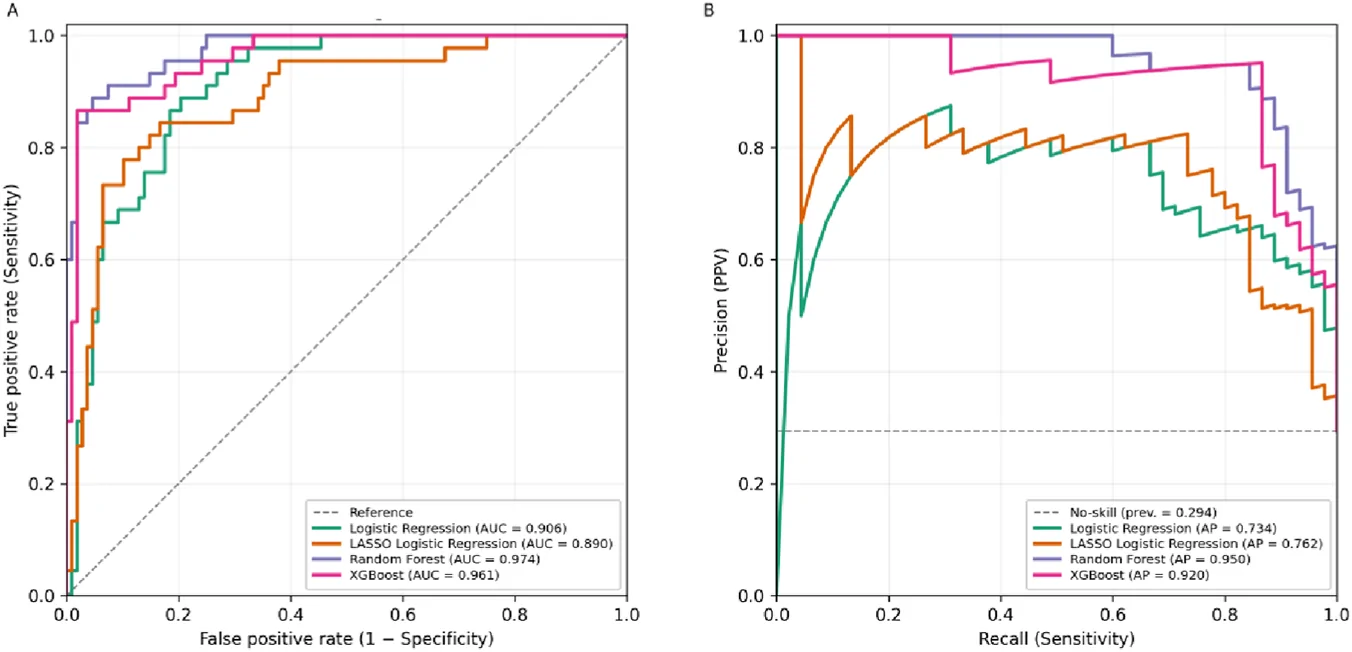
ROC and PR curves of machine learning models. **(A)** ROC curves of LR, LASSO logistic regression, RF, and XGBoost in the test cohort, with AUROC values annotated. **(B)** Precision-recall curves of the four models in the test cohort, with AUPRC values annotated.

The receiver operating characteristic (ROC) and precision-recall (PR) curves for the 4 ML models in the test set are shown in Figure 3, and detailed performance metrics are summarized in Table 3.

**TABLE 3:** Predictive performance of machine learning models and clinical scores for 28-day mortality in the test cohort.

Model	AUROC (95% CI)	AUPRC (95% CI)	Brier (95% CI)	Sensitivity	Specificity	PPV	NPV	F1
ML models								
LR	0.906 (0.849–0.948)	0.734 (0.590–0.882)	0.123 (0.090–0.163)	73.30%	86.10%	68.80%	88.60%	0.71
LASSO LR	0.890 (0.828–0.942)	0.762 (0.634–0.888)	0.131 (0.095–0.169)	82.20%	84.30%	68.50%	91.90%	0.747
RF	0.974 (0.953–0.992)	0.950 (0.908–0.984)	0.065 (0.046–0.084)	84.40%	98.10%	95.00%	93.80%	0.894
XGBoost	0.961 (0.927–0.989)	0.920 (0.869–0.963)	0.110 (0.079–0.143)	86.70%	93.50%	84.80%	94.30%	0.857
Clinical scores								
SOFA	0.677 (0.582–0.771)	0.501 (0.373–0.644)	0.223 (0.202–0.243)	46.70%	78.70%	47.70%	78.30%	0.472
SAPS II	0.791 (0.706–0.864)	0.645 (0.511–0.779)	0.187 (0.165–0.210)	73.30%	69.40%	50.00%	86.00%	0.595
APS III	0.844 (0.777–0.900)	0.637 (0.490–0.783)	0.170 (0.149–0.192)	60.00%	85.20%	62.80%	83.90%	0.614
MELD	0.804 (0.724–0.877)	0.691 (0.548–0.829)	0.181 (0.158–0.204)	53.30%	91.70%	72.70%	82.40%	0.615
OASIS	0.677 (0.572–0.782)	0.535 (0.387–0.682)	0.219 (0.194–0.244)	46.70%	85.20%	56.80%	79.80%	0.512

Among the 4 ML models, RF achieved the best overall predictive performance. It had the highest AUROC of 0.974 (95% CI: 0.953–0.992), the highest AUPRC of 0.950 (95% CI: 0.908–0.984), and the lowest Brier score of 0.065 (95% CI: 0.046–0.084), indicating strong discrimination and favorable probability estimation. At the Youden-index threshold of 0.544, the RF model achieved a sensitivity of 84.4%, specificity of 98.1%, PPV of 95.0%, NPV of 93.8%, and F1 score of 0.894.

XGBoost also showed strong performance, with an AUROC of 0.961 (95% CI: 0.927–0.989) and an AUPRC of 0.920. The two linear models achieved acceptable discrimination but were less accurate than the ensemble-based models. Specifically, LR had an AUROC of 0.906, whereas LASSO logistic regression had an AUROC of 0.890.

All 4 ML models outperformed the conventional clinical scoring systems. Among the traditional scores, APS III showed the highest discrimination, with an AUROC of 0.844. In contrast, SOFA and OASIS showed relatively limited predictive ability, both with an AUROC of 0.677.

### 3.5 Comparison with clinical scores: ROC/PR curves

**FIGURE 4:**
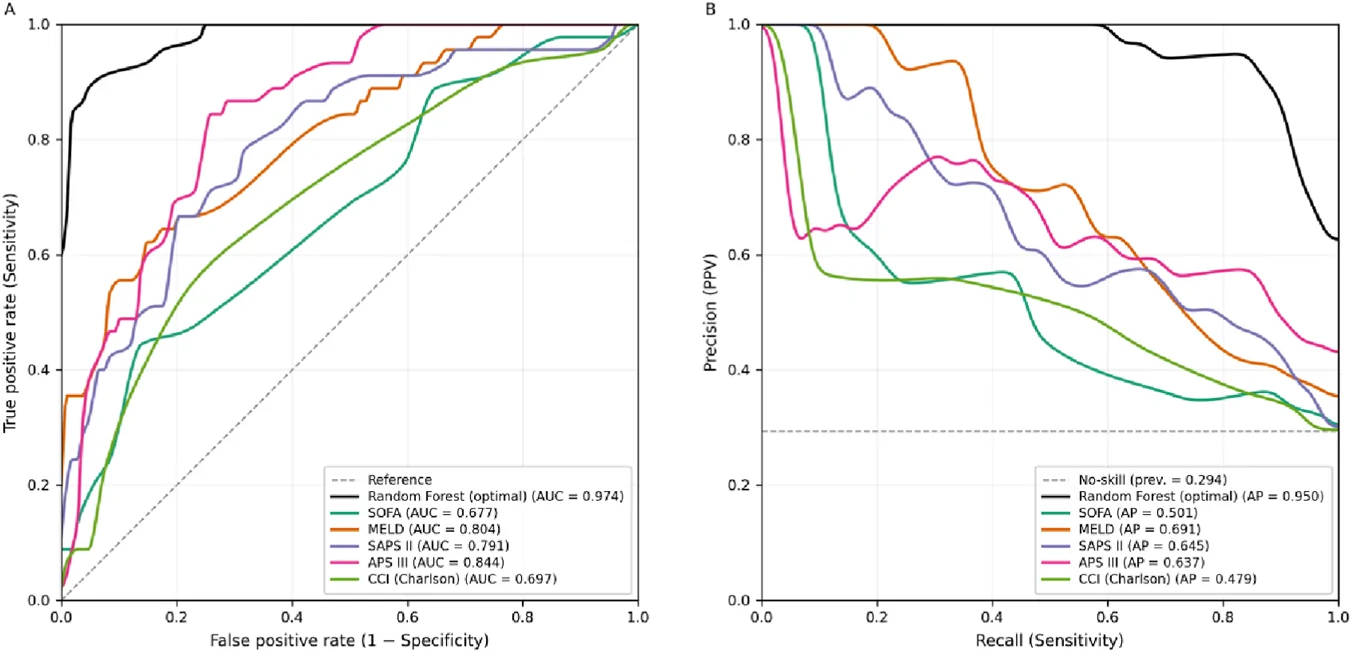
Comparison of the optimal model with conventional clinical scores. **(A)** ROC curves of RF and clinical scores, including SOFA, MELD, SAPS II, APS III, and CCI, in the test cohort. **(B)** Precision-recall curves of RF and clinical scores in the test cohort.

Figure 4 compares the RF model with conventional clinical scores, including SOFA, MELD, SAPS II, APS III, and CCI. The RF model consistently outperformed all clinical scores in both ROC and PR analyses. Although APS III achieved the best ROC performance among the conventional scores, with an AUROC of 0.844, its AUPRC was only 0.637. This suggests that conventional scores may have limited ability to maintain high precision when identifying patients at elevated mortality risk, particularly in the setting of an imbalanced outcome distribution.

### 3.6 Calibration and decision curve analysis

**FIGURE 5:**
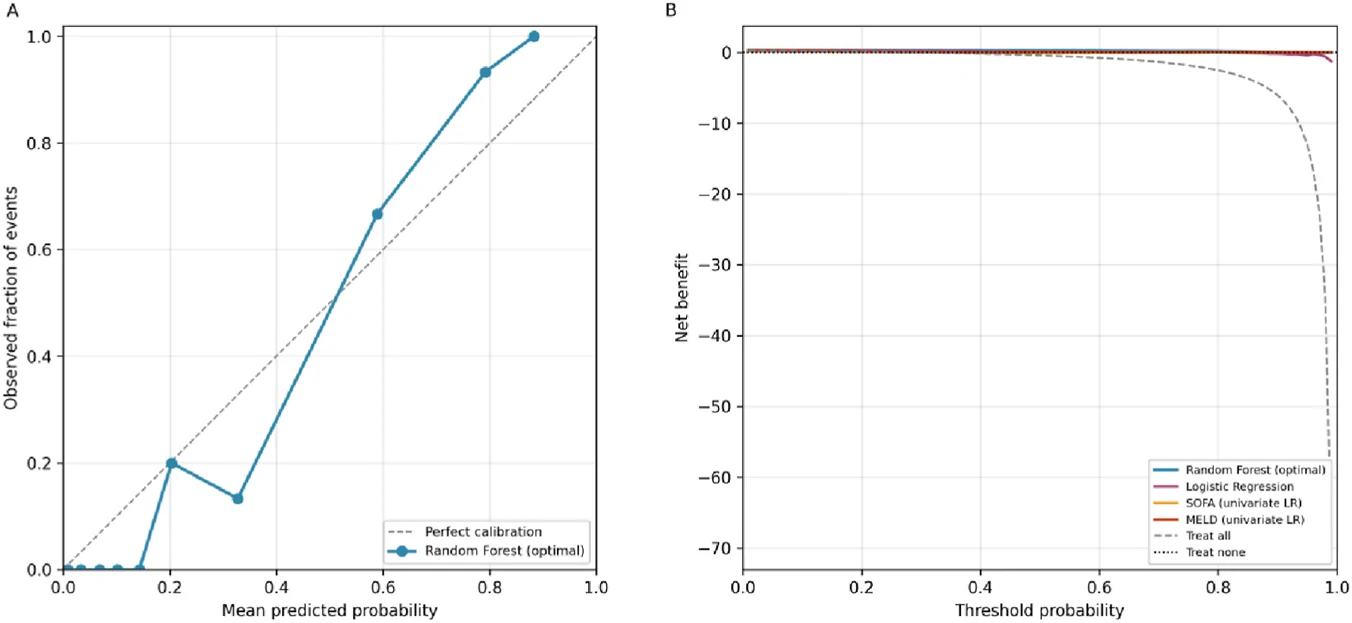
Calibration curve and decision curve analysis of the RF model. **(A)** Calibration curve comparing predicted and observed 28-day mortality. **(B)** Decision curve analysis showing the net benefit of RF, LR, SOFA, MELD, treat-all, and treat-none strategies.

The calibration curve and decision curve analysis for the RF model are presented in Figure 5. The RF model showed acceptable overall calibration, with a calibration slope of 1.806 and an intercept of 0.040, although a tendency toward overestimation was observed in the higher predicted probability range. Decision curve analysis demonstrated that RF provided greater net benefit than SOFA, MELD, and the treat-all or treat-none strategies across most clinically relevant threshold probabilities from 0.05 to 0.50. These findings suggest that the RF model may offer additional clinical value for early identification of ICU patients with liver cancer who are at high risk of 28-day mortality.

### 3.7 Model interpretability

**FIGURE 6:**
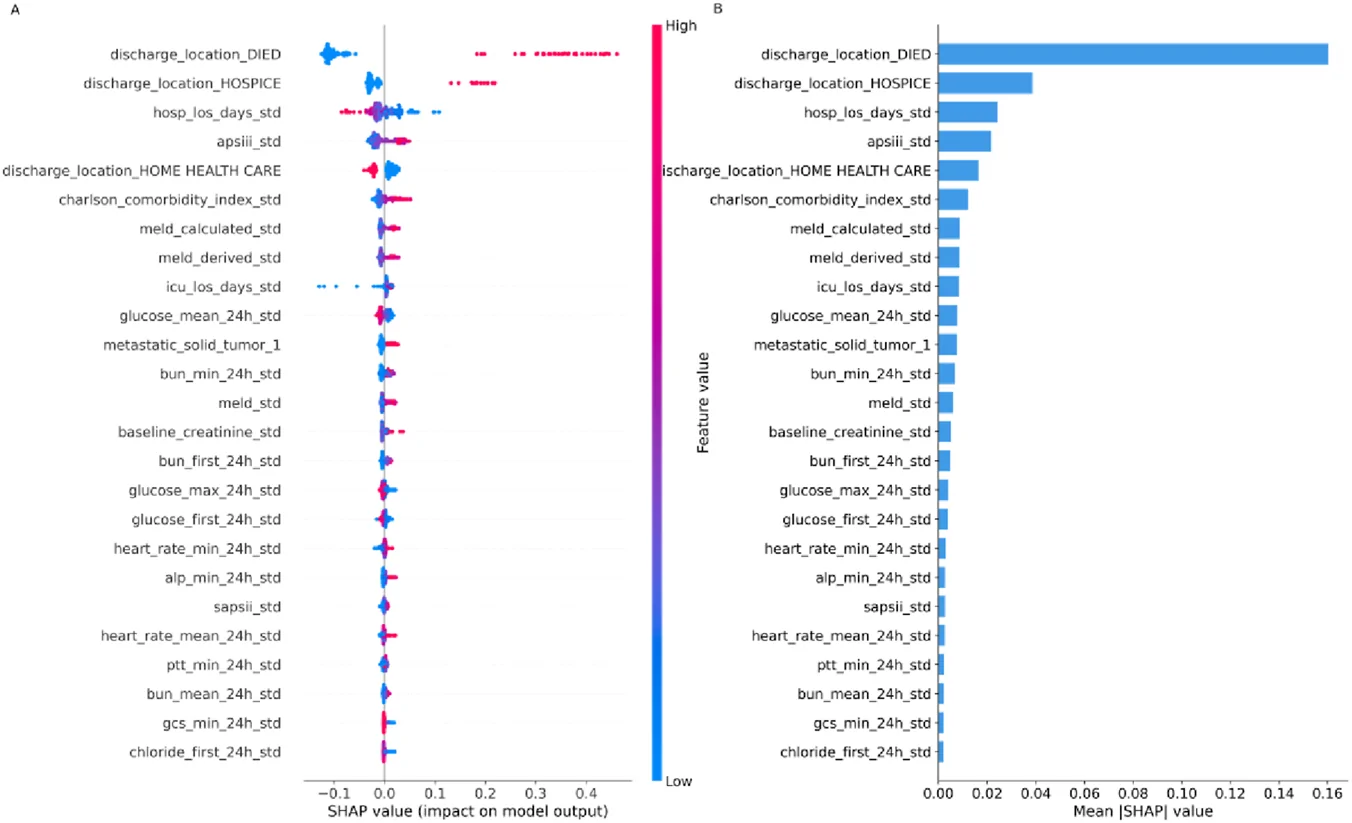
SHAP summary plot of the optimal model. **(A)** Beeswarm plot showing SHAP values of the top predictors for patients in the test set; each dot represents one patient, and color indicates the magnitude of the feature value. **(B)** Bar plot showing mean absolute SHAP values and global feature importance ranking.

SHAP summary and feature importance plots are shown in Figure 6. After excluding outcome-adjacent discharge-related variables, such as discharge disposition, the most influential clinical predictors ranked by mean absolute SHAP values included APS III, CCI, MELD and its derived components, mean and initial glucose, metastatic solid tumor status, BUN, and creatinine. These predictors represent acute physiological disturbance, long-term comorbidity burden, hepatic dysfunction, metabolic alteration, tumor burden, and renal impairment. The SHAP-based ranking was therefore clinically interpretable and broadly consistent with the observed baseline differences and Cox regression findings.

**FIGURE 7:**
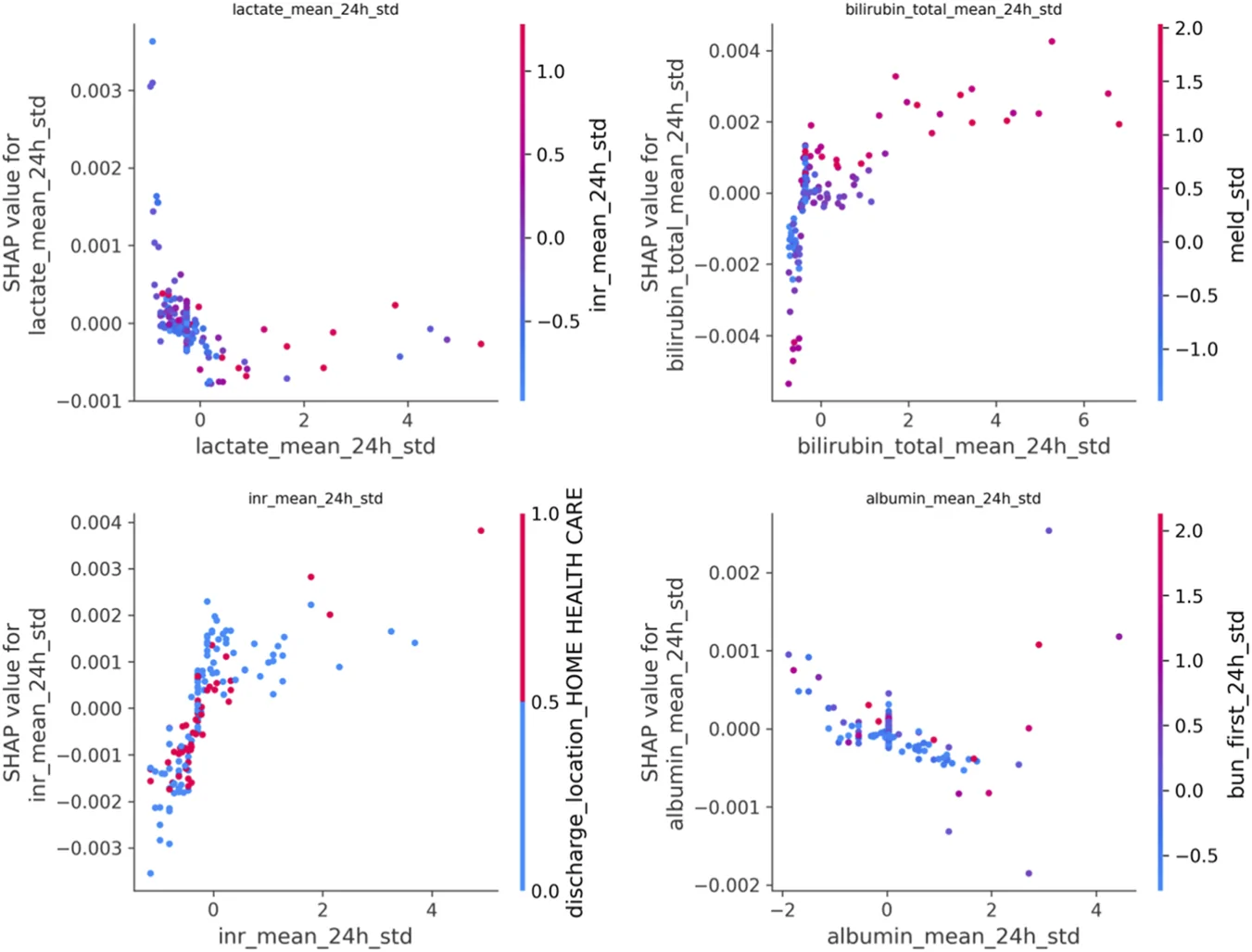
SHAP dependence plots for key predictors. Four subplots showing the relationship between feature value and SHAP value for APS III, MELD, glucose (mean), and BUN; color indicates the value of the most interacting feature.

**FIGURE 8:**
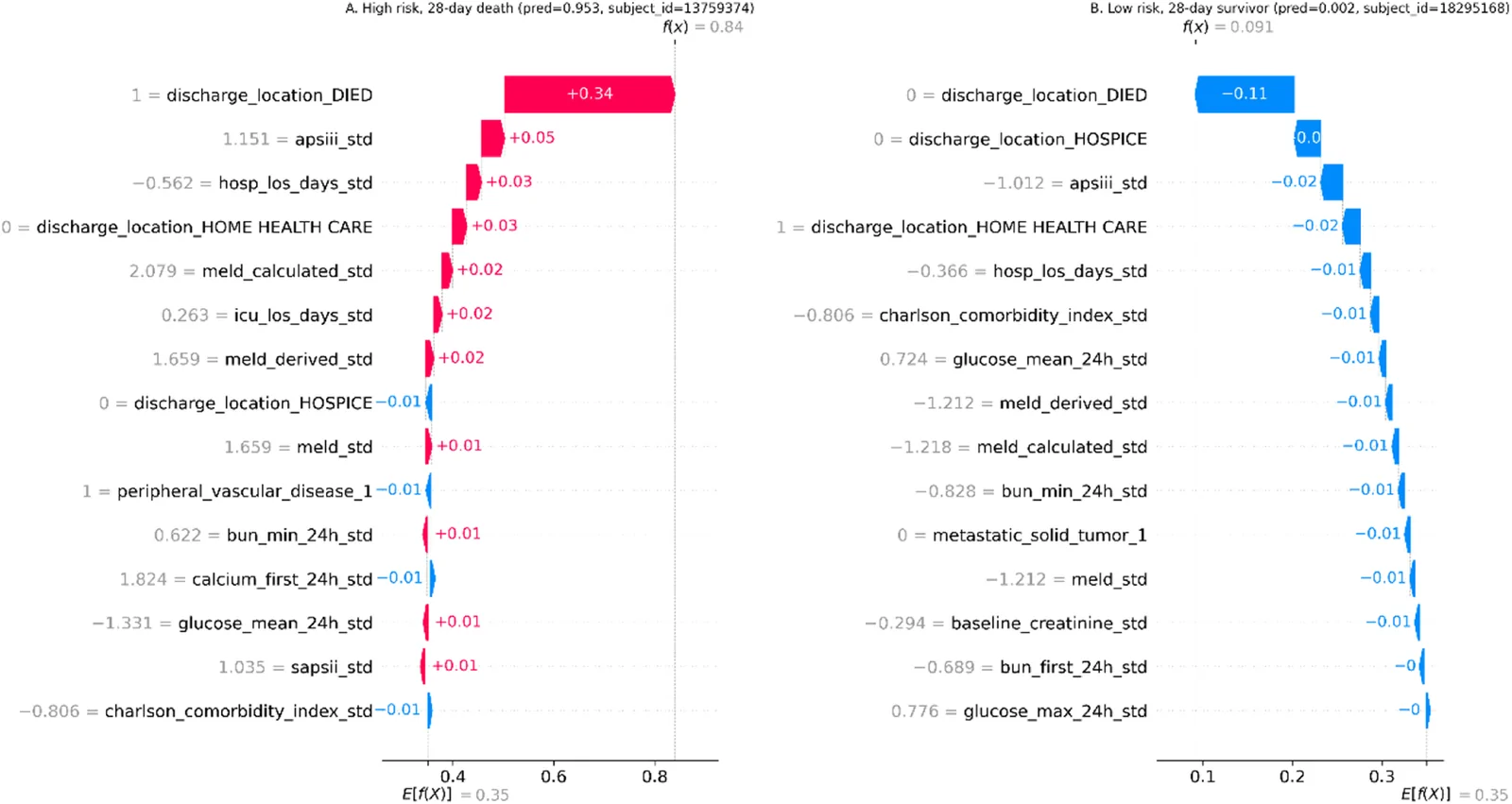
Individual SHAP waterfall plots for representative patients. **(A)** A high-risk patient who died within 28 days. **(B)** A low-risk patient who survived beyond 28 days. Each bar shows the additive contribution of individual features to the final predicted probability.

SHAP dependence plots are shown in Figure 7. Higher APS III and MELD values were associated with increased predicted mortality risk. Elevated BUN was also linked to higher predicted risk, whereas lower glucose levels tended to contribute to higher mortality predictions. Individual SHAP waterfall plots for two representative patients are presented in Figure 8, illustrating how different clinical features contributed additively to patient-level risk estimation.

### 3.8 Simplified model

Based on the SHAP feature importance ranking shown in Figure 6, a simplified 12-variable model was developed. The retained predictors included APS III, CCI, MELD-related variables, mean, first, and maximum glucose values, metastatic solid tumor status, minimum BUN, and baseline creatinine. All 12 selected predictors are routine clinical indicators that can be obtained at ICU admission or within the first 24 h after ICU admission, supporting the clinical practicability of the simplified model. The simplified model achieved an AUROC of 0.890 (95% CI: 0.835–0.934), an AUPRC of 0.720, and a Brier score of 0.123. Although its discrimination was lower than that of the full RF model, with a ΔAUROC of 0.084, it still substantially outperformed conventional clinical scores, as shown in Table 4.

**TABLE 4:** Comparison of full model, simplified model, and conventional clinical scores on the test set.

Model	AUROC (95% CI)	AUPRC	Brier	Calibration slope	Calibration intercept
RF (full)	0.974 (0.951–0.992)	0.950	0.065	1.806	0.040
Simplified model	0.890 (0.835–0.934)	0.720	0.123	1.104	0.113
SOFA	0.677 (0.587–0.769)	0.501	0.223	0.977	−0.855
MELD	0.804 (0.724–0.877)	0.691	0.181	1.455	−0.875
SAPS II	0.791 (0.706–0.864)	0.645	0.187	1.127	−0.876
APS III	0.844 (0.777–0.900)	0.637	0.170	1.132	−0.829

**FIGURE 9:**
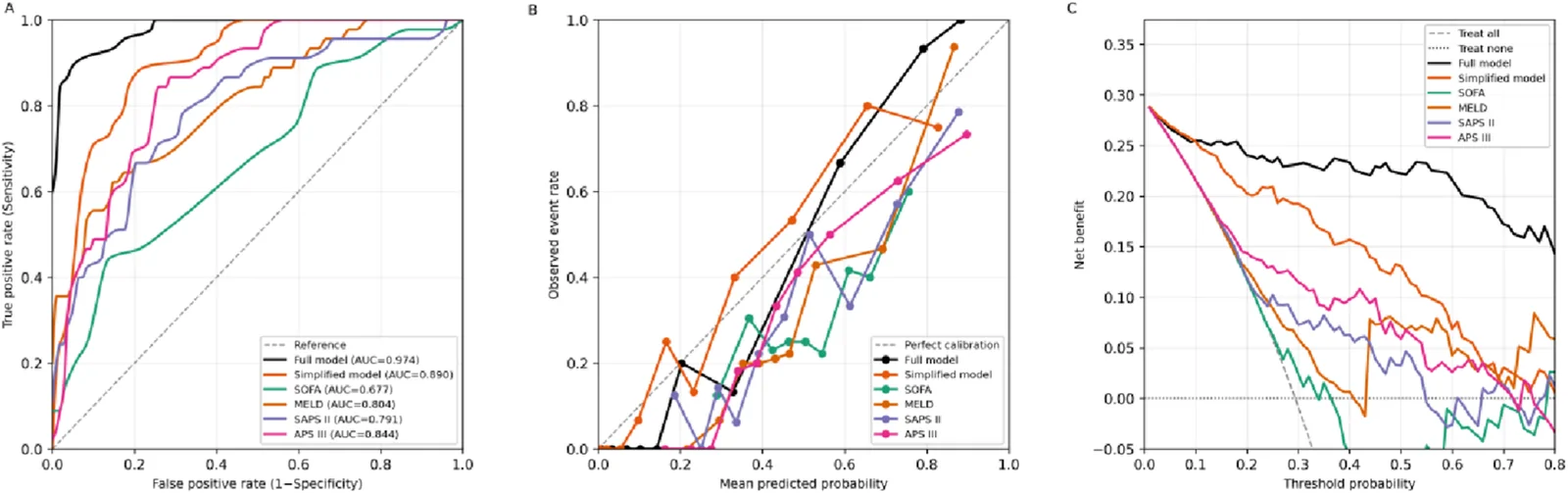
Comparison between the full model and simplified model. **(A)** ROC curves of RF full model and simplified model in the test cohort. **(B)** Calibration curves of the two models. **(C)** Decision curve analysis comparing net benefit of the full model, simplified model, SOFA, and MELD.

The comparative performance of the full RF model and the simplified model across ROC, calibration, and decision curve analyses is shown in Figure 9.

### 3.9 Kaplan-Meier survival analysis

**FIGURE 10:**
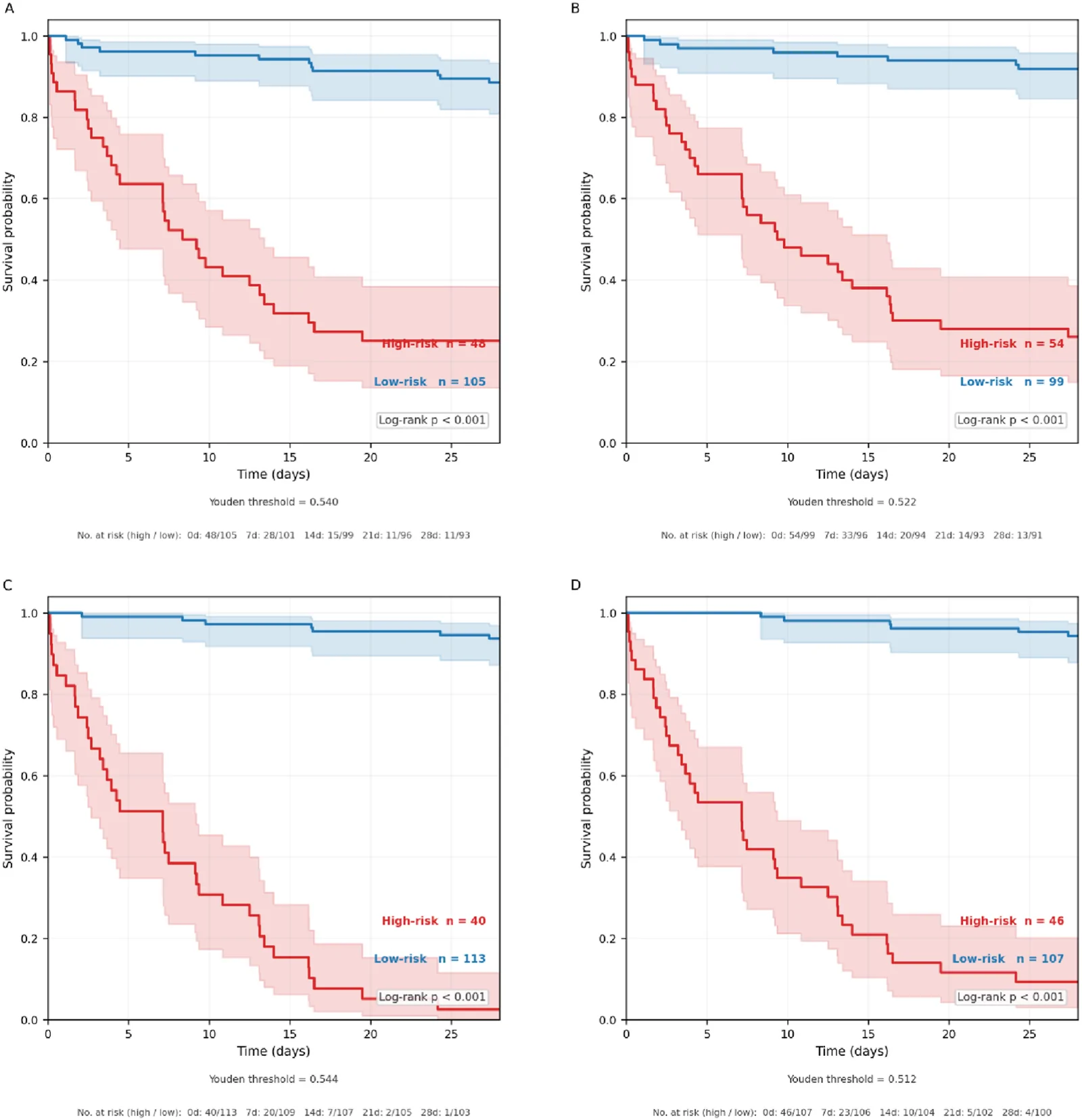
Kaplan–Meier survival curves for high-risk and low-risk groups. Four panels **(A–D)** show KM curves for LR, LASSO LR, RF, and XGBoost respectively. Patients are stratified by Youden-index thresholds derived from the training set. Log-rank p-values and at-risk counts are annotated below each subplot.

Kaplan-Meier survival curves stratified by model-predicted risk are shown in Figure 10. For the RF model, patients in the test set were divided into high-risk and low-risk groups using the Youden-index threshold derived from the training set, which was 0.544. Overall, 40 patients (26.1%) were classified as high risk, and 113 patients (73.9%) were classified as low risk. The high-risk group had markedly higher 28-day mortality than the low-risk group (95.0% vs. 6.2%; log-rank test statistic = 194.6, p < 0.001).

Similar survival separation was observed for the other ML models. Using model-specific thresholds, LR classified patients with a high-risk mortality rate of 68.8% *versus* 11.4% in the low-risk group (threshold = 0.540; p < 0.001). LASSO logistic regression showed a high-risk mortality rate of 68.5% *versus* 8.1% in the low-risk group (threshold = 0.522; p < 0.001). XGBoost showed a high-risk mortality rate of 84.8% *versus* 5.6% in the low-risk group (threshold = 0.512; p < 0.001). These results indicate that model-derived risk probabilities were strongly associated with short-term survival patterns.

### 3.10 Subgroup and sensitivity analyses

The performance of the RF model across predefined clinical subgroups is summarized in Supplementary Table S2. The model maintained high discrimination in the HCC subgroup (AUROC = 0.980, n = 116) and the cirrhosis subgroup (AUROC = 0.988, n = 103). Strong performance was also observed in patients without cirrhosis (AUROC = 0.948, n = 50) and in those receiving mechanical ventilation (AUROC = 0.979, n = 107). However, the sepsis subgroup included only two patients, and its performance estimate was therefore considered unstable and should be interpreted with caution.

Sensitivity analyses further supported the robustness of the RF model. After excluding patients who died within 24 h after ICU admission, the model retained an AUROC of 0.971. When the analysis was restricted to patients with primary HCC, the AUROC was 0.980. After removing composite severity score variables and retraining the model, the AUROC remained high at 0.966. Complete-case analysis yielded an AUROC of 0.978. Repeated training using five different random seeds also produced stable results, with AUROC values ranging from 0.966 to 0.969 and a mean ± SD of 0.967 ± 0.001. These findings suggest that the model performance was not driven by a single preprocessing strategy, patient subset, or random split.

## 4 Discussion

This study developed and internally evaluated ML-based models for predicting 28-day mortality among ICU patients with liver cancer using the MIMIC-IV database. By integrating early clinical variables available within the first 24 h after ICU admission, the present study compared 4 ML algorithms with conventional severity scores, interpreted the best-performing model using SHAP, and further constructed a simplified model for potential clinical application. Overall, the findings suggest that early mortality risk in this population is shaped by the combined effects of acute physiological instability, hepatic dysfunction, comorbidity burden, renal impairment, metabolic disturbance, and tumor-related disease burden.

The main finding was that the RF model achieved the strongest predictive performance, with an AUROC of 0.974, outperforming the two linear ML models and all conventional clinical scores evaluated in this study. SOFA, which is widely used to assess organ dysfunction in critically ill patients, showed limited discrimination in this cohort, with an AUROC of 0.677. APS III performed better than the other conventional scores, but its AUROC remained lower than that of RF and XGBoost. This result suggests that general ICU severity scores may not fully capture the complex risk structure of ICU patients with liver cancer. These patients often present with overlapping hepatic failure, tumor progression, systemic inflammation, renal dysfunction, and treatment-related vulnerability, which may not be adequately represented by scores developed for broader ICU populations or other disease-specific critical illness phenotypes (Escorsell et al., 2007).

This superior performance of RF may be partly explained by its high specificity and lower Brier score. In the test cohort, RF achieved a specificity of 98.1% and a Brier score of 0.065, indicating that the model showed both strong discrimination and favorable probability estimation. From a clinical perspective, high specificity may be useful when the model is used as a supportive tool to identify patients at particularly high risk, because excessive false-positive classification could lead to unnecessary escalation of monitoring or intervention. However, because this study was based on a single-center retrospective database, the observed performance should be interpreted as internal evidence and requires validation in independent cohorts before broader use.

The SHAP analysis provided clinically plausible explanations for the RF model. The most influential predictors included APS III, CCI, MELD, glucose-related variables, metastatic solid tumor status, BUN, and creatinine. These variables reflect several interconnected biological processes relevant to critically ill patients with liver cancer. APS III represents acute physiological derangement; CCI captures the cumulative burden of chronic illness; MELD reflects liver synthetic and excretory dysfunction; and BUN and creatinine indicate renal dysfunction, hypoperfusion, or catabolic stress. The contribution of metastatic solid tumor status further suggests that tumor burden and systemic cancer progression are important components of early ICU mortality risk. Together, these findings indicate that mortality prediction in this population depends not on a single organ system but on the interaction between hepatic reserve, tumor-related burden, and systemic organ failure.

Another clinically relevant finding from the SHAP analysis was the association between lower glucose values and increased predicted mortality. In ICU patients with liver cancer, low or relatively reduced glucose may reflect impaired hepatic gluconeogenesis, depleted metabolic reserve, malnutrition, cancer-related cachexia, or advanced systemic illness. This differs from the more familiar pattern in some general ICU populations, where stress hyperglycemia is often viewed as a marker of severity. In patients with advanced liver disease or liver cancer, however, the loss of hepatic metabolic buffering capacity may make glucose dysregulation more complex and clinically meaningful. Similarly, the importance of BUN is biologically reasonable, as elevated BUN may represent renal hypoperfusion, protein catabolism, gastrointestinal bleeding, or worsening multi-organ dysfunction in critical illness (Macedo et al., 2011).

The Cox regression analysis provided complementary evidence to the ML findings. Liver failure, lactate, albumin, INR, platelet count, BUN, MELD, and CCI remained independently associated with 28-day mortality in the multivariate model. These factors are closely related to hepatic synthetic function, coagulation disturbance, tissue hypoperfusion, renal impairment, and baseline comorbidity. Albumin showed a protective association, which is consistent with its role as a marker of hepatic synthetic reserve, nutritional status, systemic inflammation, and vascular homeostasis (Cabrerizo et al., 2015). Although vasopressor use was significant in univariate analysis, it was no longer significant after multivariate adjustment. This attenuation may indicate that the prognostic information carried by vasopressor use overlaps with broader severity indicators, such as liver failure, lactate elevation, and MELD score.

The Kaplan-Meier analysis further supported the risk stratification ability of the ML-derived probabilities. Patients classified as high risk by the RF model had markedly higher 28-day mortality than those classified as low risk. Similar survival separation was also observed for LR, LASSO logistic regression, and XGBoost, suggesting that the predicted probabilities were not merely statistical outputs but were meaningfully associated with short-term survival patterns. Nevertheless, these findings should be interpreted as evidence of prognostic stratification rather than proof that model-guided interventions would improve outcomes. Future studies are needed to evaluate whether incorporating such models into clinical workflows can improve decision-making, communication, or allocation of monitoring resources.

The simplified 12-variable model retained good predictive performance, with an AUROC of 0.890, while using a smaller set of clinically accessible variables. Although the simplified model was less accurate than the full RF model, it still outperformed conventional severity scores in this cohort. This trade-off may be important for practical implementation, especially in clinical environments where automated calculation of complex ICU scores is not routinely available. The simplified model may therefore serve as a more feasible risk estimation tool, but its calibration and transportability should be tested in external datasets before use in routine clinical practice.

Several limitations should be considered. First, this was a single-center retrospective study based on MIMIC-IV, a database from a US academic medical center. Therefore, the findings may not be directly generalizable to other healthcare systems or regions, particularly where the etiological distribution of liver cancer differs, such as hepatitis B-dominant populations in Asia or alcohol- and metabolic dysfunction-associated liver disease-dominant populations in Western countries (McGlynn et al., 2021). Second, although the cohort included 507 patients and 150 deaths, the sample size remained relatively modest for ML model development, and some subgroup analyses were limited by small sample sizes. In particular, the sepsis subgroup was too small to support reliable performance estimation. Third, the study period of MIMIC-IV covers years during which liver cancer treatment strategies changed substantially, including the increasing use of immunotherapy, tyrosine kinase inhibitors, and liver-directed therapies. These treatment-related variables were not fully captured in the current analysis, which may have introduced residual confounding. Fourth, patients with decompensated cirrhosis and acute-on-chronic liver failure may represent distinct syndromic contexts in which organ failure scores behave differently, and this heterogeneity was not fully resolved in the present analysis (Moreau et al., 2013; Duseja et al., 2013). Fifth, some highly ranked SHAP features in the full model were close to outcome-related processes. Although variables with potential leakage were excluded when constructing the simplified model, this issue highlights the need for careful variable selection when translating retrospective models into prospective settings. Finally, SHAP improves model transparency but does not establish causal relationships. The observed feature contributions should therefore be interpreted as model-based associations rather than direct mechanistic effects. External validation in independent cohorts is required before clinical implementation (Steyerberg and Harrell, 2016).

Despite these limitations, this study provides an interpretable ML framework for early 28-day mortality prediction in ICU patients with liver cancer. The results suggest that integrating acute physiological scores, liver-specific severity markers, metabolic variables, renal function indicators, comorbidity burden, and tumor-related information may improve risk stratification in this high-risk population. More importantly, the SHAP-based interpretation highlights the multi-system nature of mortality risk in critically ill patients with liver cancer, linking hepatic dysfunction, metabolic instability, renal impairment, and systemic illness severity. Future multicenter studies with prospective validation, treatment-related variables, and dynamic longitudinal data are needed to confirm the robustness of this approach and to determine whether ML-assisted risk assessment can support clinical decision-making in real-world ICU settings.

## 5 Conclusion

In this retrospective cohort study based on MIMIC-IV, we developed and internally evaluated an RF-based ML model for predicting 28-day mortality among ICU patients with liver cancer. The RF model achieved excellent discrimination, with an AUROC of 0.974, and outperformed both linear ML models and conventional clinical scoring systems. SHAP analysis identified APS III, CCI, MELD, glucose-related variables, BUN, creatinine, and metastatic solid tumor status as major contributors to model prediction, suggesting that early mortality risk in this population is closely related to acute physiological derangement, hepatic dysfunction, metabolic instability, renal impairment, comorbidity burden, and tumor-related disease severity.

A simplified 12-variable model retained good predictive accuracy and may provide a more practical option for early risk assessment when complete model inputs are not readily available. Kaplan-Meier analysis further showed clear survival separation between model-defined high-risk and low-risk groups. These findings suggest that interpretable ML models may help support early prognostic assessment in critically ill patients with liver cancer. However, external validation in multicenter cohorts and prospective evaluation are required before clinical implementation.

## Data Availability

The original contributions presented in the study are included in the article/Supplementary Material, further inquiries can be directed to the corresponding authors.
